# The role of angiogenic factors and their soluble receptors in acute lung injury (ALI)/ acute respiratory distress syndrome (ARDS) associated with critical illness

**DOI:** 10.1186/1476-9255-10-6

**Published:** 2013-02-11

**Authors:** Takeshi Wada, Subrina Jesmin, Satoshi Gando, Yuichiro Yanagida, Asumi Mizugaki, Sayeeda Nusrat Sultana, Sohel Zaedi, Hiroyuki Yokota

**Affiliations:** 1 Division of Acute and Critical Care Medicine, Department of Anesthesiology and Critical Care Medicine, Hokkaido University Graduate School of Medicine, N17W5, Kita-ku, Sapporo, Hokkaido, 060-8638, Japan; 2 Deparment of Emergency and Critical Care Medicine, Faculty of Medicine, University of Tsukuba, 1-1-1, Tennoudai, Tsukuba, Ibaraki, 305-8575, Japan; 3 Health and Diseases Research Center for Rural Peoples (HDRCRP), 14/15, 1st floor, Probal Housing Ltd., Shekertak (Adjacent to Shekertak Road 1), Mohammadpur, Dhaka, 1207, Bangladesh; 4 Department of Emergency and Critical Care Medicine, Nippon Medical School, 1-1-5 Sendagi Bunkyo-ku, Tokyo, 113-8603, Japan

**Keywords:** Acute lung injury, Acute respiratory distress syndrome, Angiogenic factors, Vascular endothelial growth factor, Angiopoietin, Outcome

## Abstract

**Background:**

Acute lung injury (ALI) and acute respiratory distress syndrome (ARDS) are characterized by a disruption of the endothelium and alveolar epithelial barriers involving increased microvascular permeability, thus resulting in the set of protein-rich pulmonary edema. Angiogenic factors and their receptors, including vascular endothelial growth factor (VEGF)/VEGF-receptor (VEGFR) and the angiopoietin (Ang)/Tie2 signaling pathways, play pivotal roles in both angiogenesis and microvascular permeability. The aim of the study was to assess the relationship between angiogenic factors, their soluble receptors and ALI/ARDS associated with critically ill patients, including sepsis, severe trauma, and post-cardiac arrest syndrome (PCAS).

**Methods:**

One hundred fifty-nine critically ill patients, including 50 patients with sepsis, 57 patients with severe trauma and 52 resuscitated after out-of-hospital cardiac arrest, were divided into three subgroups: including 25 ALI patients, 101 ARDS patients and 22 non-ALI/ARDS patients. The serum levels of angiogenic factors were measured at the time of admission (day 1), as well as day 3 and day 5 and then were compared among the ALI, ARDS and non-ALI/ARDS groups. Their predictive values for developing ALI/ARDS and 28-day mortality were evaluated.

**Results:**

Higher levels of sVEGFR1 and Ang2 were observed in the ALI and ARDS patients than in the non-ALI/ARDS patients during the entire study period. The Ang2/Ang1 ratio in the ARDS group was also significantly higher than that in the non-ALI/ADRS group. The sVEGFR2 levels in the ARDS group on day 1 were significantly lower than those of the non-ALI/ADRS group. In addition, significant positive correlations were seen between the sVEGFR1, Ang2, Ang2/Ang1, and the development of ALI/ARDS in critical illness. There were also significant negative correlations between the minimal value of sVEGFR2, the maximal value of Ang1 and the ALI/ARDS group. In particular, sVEGFR2 and Ang2 were independent predictors of developing ALI/ARDS. Moreover, Ang2 and sVEGFR2 also independently predicted the mortality in ALI/ARDS patients.

**Conclusions:**

Angiogenic factors and their soluble receptors, particularly sVEGFR2 and Ang2, are thus considered to be valuable predictive biomarkers in the development of ALI/ARDS associated with critical illness and mortality in ALI/ARDS patients.

## Background

Acute lung injury (ALI) and its most severe manifestation, acute respiratory distress syndrome (ARDS), are clinically defined as a severe dysfunction of gas exchange and chest radiographic abnormalities in the absence of heart failure [1,2]. ALI/ARDS are devastating complications of numerous severe conditions, including severe sepsis, severe trauma, and ischemia/reperfusion injury. ALI/ARDS are characterized by a disruption of the endothelium and alveolar epithelial barriers involving increased microvascular permeability, thus resulting in the set of protein-rich pulmonary edema [3,4]. Novel mediators that may be involved in ALI/ARDS are angiogenic factors, including vascular endothelial growth factor (VEGF)/VEGF receptor (VEGFR) signaling pathway and the angiopoietin(Ang)/Tie2 signaling pathway.

VEGF is a glycoprotein that is synthesized and released by vascular endothelial cells, lung epithelium, platelet, and leukocytes [5]. VEGF can enhance angiogenesis and increase microvascular permeability through binding with the VEGFR, which may thus lead to edema and hypotension [5,6]. VEGF mainly binds to two transmembrane receptors, VEGF receptor-1 (VEGFR1) and VEGFR2. VEGFR2, which is selectively expressed in the endothelium, mainly mediates endothelial growth, survival signals, proliferation and permeability and pathological angiogenesis. In contrast, VEGFR1, which is present both on endothelial cells and monocytes, plays an important role by increasing the vascular permeability under pathological conditions, such as ischemia and inflammation.

The angiopoietin (Ang)-Tie2 ligand-receptor system is restricted to the regulation of the endothelium and it is also involved in multiple organ dysfunction-related pathways [7]. The Ang-Tie2 system not only regulates angiogenesis, but it also controls endothelial inflammation, along with VEGF and its receptor system [8]. Ang1 stabilizes the endothelial cells, inhibits vascular leakage, and suppresses inflammatory and coagulation-related gene expression through Tie2 activation [8-10]. Ang2 antagonizes the binding of Ang1 to Tie2. Therefore, Ang2 is thought to act as a proinflammatory mediator increasing fluid leakage through the endothelial vasculature [11]. However, Ang2 promotes cell survival in the presence of VEGF [12]. Several studies have demonstrated the ratio of Ang1 to Ang2 to better describe the state of activation of the endothelium, because Ang1 and Ang2 have agonist–antagonist properties on the endothelium [13,14].

This study investigated the hypothesis that the angiogenic factors play pivotal roles in the development of ALI/ARDS, thus leading to a poor prognosis in critically ill patients. The present study examined the serial changes in serum angiogenic factors and their soluble receptors in ALI/ARDS associated with critical illness, including severe trauma, sepsis, and PCAS. The study also investigated the relationships between these factors and the development of ALI/ARDS and their mortality.

## Methods

### Patients

Approval for this study was obtained from the institutional review board, the Ethics Committee of Hokkaido University School of Medicine. Informed consent for this study was obtained from the patients’ next of kin. Systemic inflammatory response syndrome (SIRS), sepsis, severe sepsis, and septic shock were defined according to the American College of Chest Physicians/Society of Critical Care Medicine consensus conference as previously published [15]. Infection was defined as any localization with clinical evidence of infection and the identification of microorganisms grown from bacteriological samples. Severe trauma patients were defined as those with an Injury Severity Score (ISS) ≥ 9 (at least one abbreviated Injury Scale ≥ 3) [16]. Cardiac arrest was defined as the absence of a palpable pulse confirmed by an emergency medical service. Cardiopulmonary resuscitation was performed in accordance with the Guidelines 2000 for Cardiopulmonary Resuscitation and Emergency Cardiovascular Care [17]. Fifteen healthy volunteers served as the control subjects. The patient sources were the same as that described in our previous studies (severe trauma; [18], sepsis; [19], PCAS; [20]).

### Definitions

ARDS was defined based on the American-European Consensus Conference on ARDS [1].The severity of illness of the patients was evaluated according to the Acute Physiology and Chronic Health Evaluation (APACHE) II score at the time of enrollment [21]. Organ dysfunction was assessed by the Sequential Organ Failure Assessment (SOFA) score within 24 hr after arrival at the emergency department (day1), as well as on days 3 and 5 [22]. All patients received mechanical ventilation in a pressure-controlled and pressure support ventilation mode with a positive end-expiratory pressure. Ventilator management was performed based on a lung protective strategy as designed by the ARDS network [23]. We defined the maximum score (max) or minimum score (min) as the highest or lowest score on day1, day3 and day5, when we collected the patient data and samples.

### Study protocol and measurement methods

Blood samples were collected by an arterial catheter within 12 hours after arrival at the emergency department (day1), as well as on days 3 and 5. The blood was immediately placed into individual tubes and centrifuged at 3,000 rpm, for 5 min at 4°C. The serum samples were stored at −80°C until used for the assay.

The following variables were measured in duplicate: VEGF (Quantikine; R&D systems, Inc. Human VEGF, Minneapolis, MN, USA); sVEGFR1 (Quantikine; R&D systems, Inc. Human sVEGF R1/Flt-1, Minneapolis, MN, USA); sVEGFR2 (Quantikine; R&D systems, Inc. Human sVEGF R2/KDR/Flk-1, Minneapolis, MN, USA); Ang1 (Quantikine; R&D systems, Inc. Human Angiopoietin-1, Minneapolis, MN, USA); Ang2 (Quantikine; R&D systems, Inc. Human Angiopoietin-2, Minneapolis, MN, USA); and soluble Tie2 receptor (sTie2) (Quantikine; R&D systems, Inc. Human Tie-2, Minneapolis, MN, USA).

### Statistical analysis

The statistical analyses and calculations were performed with the SPSS 19.0 software package (SPSS, Inc, Chicago, IL, USA). Differences between the two groups were analyzed using a two-sided nonparametric Mann–Whitney U test, and categorical variables were compared using Pearson’s chi-square test or Fisher’s exact test when required. The Kruskal-Wallis analysis of variance was used to compare the 3 groups. A stepwise logistic regression analysis was used to assess the relationship between the development of ALI/ARDS and the age, gender, APACHE II score, SIRS max, VEGF min, sVEGFR2 max, sVEGFR2 min, Ang1 min, Ang2 max, Ang2/Ang1 max, and sTie2 max by referencing the results of any serial changes for all angiogenic factors. A stepwise logistic regression analysis was also performed to assess the relationship between the 28-day mortality in ALI/ARDS patients and the same parameters. Variables found to be statistically significant at a 10% level according to a univariate analysis were included in the multivariable model. The results of the regression were reported as the odds ratio (OR) and 95% confidence intervals (CI). A p-value < 0.05 was considered to be statistically significant. All results were expressed as the means ± SEM, unless otherwise stated.

## Results

### Patients’ characteristics

One-hundred-fifty-nine critically ill patients, including 50 patients with sepsis, 57 patients with severe trauma and 52 resuscitated out-of hospital cardiac arrest patients, were divided into three subgroups: including 25 ALI patients, 101 ARDS patients and 22 non-ALI/ARDS patients. The baseline characteristics of the patients are shown in Table 1. ALI and ARDS groups had significantly higher APACHE II scores (p = 0.003) and maximal SOFA scores (p < 0.001), although the maximal SIRS scores was not significantly different among three groups (p = 0.172). The outcome did not achieve the significant difference among three groups.

**Table 1:** The baseline clinical characteristics of ALI, ARDS and non-ALI/ARDS patients

	**Non-ALI/ARDS**	**ALI**	**ARDS**	**p-value**
	**(n = 33)**	**(n = 25)**	**(n = 101)**	
Primary disease				
sepsis/trauma/PCAS	3/19/11	7/7/11	40/31/30	0.007
Age (years)	49.3 ± 3.8	56.4 ± 4.0	58.2 ± 1.9	0.077
Gender (male/female)	22/11	11/14	64/37	0.156
APACHE II score	20.1 ± 1.2	27.2 ± 1.5	26.8 ± 0.9	0.003
SOFA score max	4.6 ± 0.3	6.1 ± 0.6	9.7 ± 0.5	<0.001
SIRS score max	3.1 ± 0.1	3.2 ± 0.2	3.4 ± 0.1	0.172
P/F ratio min	382 ± 15.1	240 ± 5.9	111 ± 4.1	<0.001
Outcome (survived/died)	27/6	16/9	66/35	0.197

ALI, acute lung injury; ARDS, acute respiratory distress syndrome; PCAS, post-cardiac arrest syndrome; APACHE, Acute Physiology and Chronic Health Evaluation; SOFA, Sequential Organ Failure Assessment; SIRS, systemic inflammatory response syndrome; P/F, PaO_2_/FiO_2_; max, maximum score; min, minimum score.

### Serial changes in angiogenic factors and their soluble receptors

**Figure 1:**
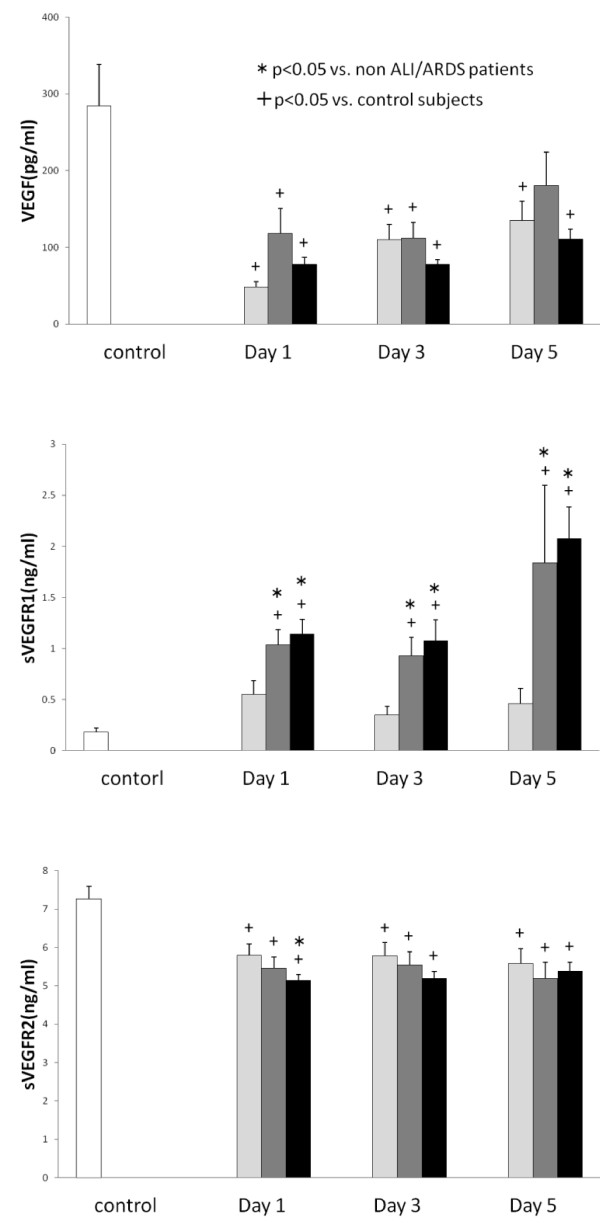
**The levels of VEGF, sVEGFR1 and sVEGFR2 in patients with critical illness.** White bars, control subjects; Light gray bars, non-ALI/ARDS patients; Dark gray bars, ALI patients; Black bars, ARDS patients.

**Figure 2:**
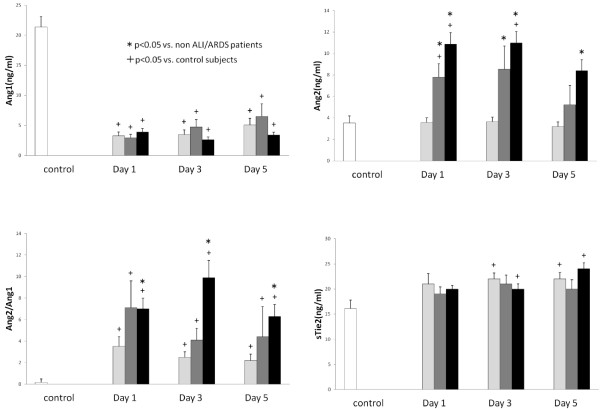
**The levels of Ang1, Ang2, sTie2 and the ratio of Ang2/Ang1 in patients with critical illness.** White bars, control subjects; Light gray bars, non-ALI/ARDS patients; Dark gray bars, ALI patients; Black bars, ARDS patients.

Serial changes in the circulating VEGF, sVEGFR1, and sVEGFR2 levels are presented in Figure 1. The VEGF levels showed no significant differences between non-ALI/ALDS and ALI/ARDS groups. Although the sVEGFR1 levels did not differ between non-ALI/ARDS and control groups, the sVEGFR1 levels in the ALI and ARDS groups were significantly higher than those of the control and non-ALI/ARDS groups during the entire study period (non-ALI/ARDS group vs. ALI group: day 1, p = 0.001; day 3, p = 0.001; day 5, p *=* 0.016, non-ALI/ARDS group: day 1, p = 0.001; day 3, p < 0.001; day 5, p < 0.001). All three groups showed significantly lower levels of sVEGFR2 in comparison to the controls. In particular, those in the ARDS group showed significantly lower sVEGFR2 levels on day 1 in comparison to non-ALI/ARDS subjects (p = 0.011). Figure 2 presents the Ang1, Ang2, sTie2 levels and the Ang2/Ang1 ratio. Although the Ang1 levels in all three groups were lower than those in control subjects, there were no significant differences between the non-ALI/ARDS and ALI/ARDS patients. On the contrary, the Ang2 levels in ALI/ADRS were significantly higher than those in the non-ALI/ARDS patients during the study period and the effects were more prominent in ARDS patients (non-ARDS vs. ARDS: day 1, p < 0.001; day 3, p *<* 0.001; day 5, p *<* 0.001). Therefore, the Ang2/Ang1 ratio significantly increased in ARDS patients (non-ARDS vs. ARDS: day 1, p = 0.010; day 3, p *<* 0.001; day 5, p *=* 0.001). The sTie2 levels were not significantly different between non-ALI/ADRS and ALI/ARDS groups.

### Relationships between angiogenic factors, their soluble receptors and ALI/ARDS

The following variables were found to be statistically significant at a 10% level in the univariate analysis and subjected to multivariate logistic regression analysis: age, APACHE II, minimum value of VEGF, and maximum values of sVEGFR1, Ang2, and Ang2/Ang1. Except for sVEGFR2 (p = 0.021) and Ang2 (p = 0.025), all other variables did not remain significant in the multivariable setting (Table 2).

**Table 2:** The results of the univariate and multivariate logistic regression analysis for predicting the development of ALI/ARDS in critical ill patients

	**Univariate**			**Multivariate**		
**Variables**	**OR**	**95% CI**	**P-value**	**OR**	**95% CI**	**P-value**
Age (years)	1.022	1.003-1.042	0.024	1.013	0.989-1.038	0.289
Gender (m/f)	1.360	0.607-3.046	0.455			
APACHE II	1.090	1.035-1.147	0.001	0.998	0.929-1.071	0.946
SIRS max	1.517	0.899-2.599	0.119			
VEGF min	1.012	0.998-1.025	0.093	1.010	0.990-1.031	0.336
sVEGFR1 max	1.803	1.221-2.664	0.003	1.303	0.878-1.933	0.188
sVEGFR2 min	0.763	0.601-0.968	0.026	0.675	0.484-0.941	0.021
Ang1 min	1.011	0.833-1.226	0.914			
Ang2 max	1.163	1.060-1.275	0.001	1.188	1.022-1.382	0.025
Ang2/Ang1 max	1.104	1.019-1.195	0.015	1.013	0.946-1.083	0.719
sTie2 max	0.995	0.960-1.032	0.783			

We decided the maximum or minimum values of covariates based on the results of Figure 1 and 2.

OR: odds ratio; CI: confidence interval.

### Relationships between angiogenic factors, their soluble receptors and 28-day mortality in ALI/ARDS patients

Table 3 shows that there were significant positive correlations between APACHE II, the maximum values of Ang2, Ang2/Ang1 ratio, and 28-day mortality in ALI/ARDS associated with critical illness. Negative correlations were also observed between the minimal value of sVEGFR2 and mortality. In particular, APACHE score, the maximum value of Ang2 and the minimum value of sVEGFR2 were also strong, independent prognostic factors for the 28-day mortality in ALI/ARDS associated with critical illness.

**Table 3:** The results of the univariate and multivariate logistic regression analysis for predicting the 28 day mortality in critical ill patients

	**Univariate**			**Multivariate**		
**Variables**	**OR**	**95% CI**	**P*- *value**	**OR**	**95% CI**	**P-value**
Age (years)	1.016	0.998-1.034	0.082	1.000	0.978-1.022	0.977
Gender (m/f)	0.733	0.364-1.473	0.383			
APACHE II	1.173	1.111-1.238	<0.001	1.148	1.082-1.218	<0.001
SIRS max	1.137	0.707-1.828	0.596			
VEGF min	0.999	0.989-1.009	0.852			
sVEGFR1 max	1.075	0.942-1.227	0.285			
sVEGFR2 min	0.712	0.579-0.875	0.001	0.689	0.526-0.904	0.007
Ang1 min	0.918	0.755-1.117	0.395			
Ang2 max	1.051	1.017-1.086	0.003	1.047	1.005-1.091	0.027
Ang2/Ang1 max	1.034	1.007-1.062	0.014	1.033	0.999-1.069	0.054
sTie2 max	0.989	0.952-1.027	0.549			

We decided the maximum or minimum values of covariates based on the results of Figures 1 and 2.

OR, odds ratio; CI, confidence interval.

## Discussion

The present study have demonstrated that ALI/ARDS patients associated with critical illness showed significant increases in their APACHE II scores, SOFA scores, sVEGFR1 and Ang2 levels as well as in the Ang2/Ang1 ratio, and decreases in their sVEGFR2. In particular, the maximum value of Ang2 and the minimal value of sVEGFR2 were the most prominent predictors of development of ALI/ARDS in critically ill patients. In addition, Ang2 max and sVEGFR2 min predicted the 28-day mortality in ALI/ARDS patients.

The systemic overexpression of VEGF plays a pivotal role in the development of pulmonary edema [24]. On the other hand, some animal studies and clinical data support a protective role for VEGF in ALI/ARDS [24]. Moreover, lower VEGF levels are also associated with organ dysfunction and a poor outcome in patients with sepsis [25]. Several studies have reported that the plasma VEGF levels in the patients with septic shock are higher than those of patients without shock, and that the VEGF concentration at the time of admission correlates with the severity of disease [26]. Therefore, the levels of VEGF in patients with sepsis, septic shock and other critical illnesses remain controversial [5,13,18]. Several previous studies have demonstrated plasma to be the preferred medium because the platelet-mediated secretion of VEGF during the clotting process could elevate the levels of VEGF [19,27]. Platelets are one of the main transporters of circulating VEGF, and VEGF is released from platelets by following their thrombin-induced activation. Critically ill patients are often affected by disseminated intravascular coagulation (DIC). The values of VEGF measured in serum samples may therefore decrease because of DIC induced platelet consumption.

The present study found the sVEGFR1 levels in ALI/ARDS patients to be significantly higher than those of non-ALI/ARDS patients during the entire study period. VEGF and VEGFR1 on the cell surface are up-regulated after endothelial injury, promoting reendothelialization by enhancing vascular remodeling [28]. These results suggest that higher sVEGFR1 levels may hamper VEGF signaling in the process of endothelial repair, thereby leading to disease progression and organ dysfunction [5,29]. Furthermore, high circulating sVRGFR1 levels correlate with morbidity and mortality, and are a potent marker of disease severity in septic or critically ill patients [5,26]. The current results were consistent with these previous studies.

VEGFR2 is a high-affinity receptor for VEGF and is regarded as the main signaling receptor for VEGF bioactivity, including angiogenesis, proliferation and permeability [30,31]. The present study found that the levels of sVEGFR2 in ARDS patients were lower than those of non-ALI/ARDS patients. Moreover, the decrease in sVEGFR2 predicted the development of ALI/ARDS. sVRGFR2 is a soluble truncated form of VEGFR2 [18]. The reduction of the sVEGFR2 level found in the current study might result from reduced shedding of the membrane-integrated form of VEGFR2 [32]. This result may reflect the augmentation of VEGF/VGFR2 signaling pathway, namely exacerbation of pulmonary vascular permeability, leading to ALI/ARDS. This is the first report evaluating the relationship between the sVEGFR2 and ALI/ARDS.

Previous studies indicated that lower Ang1 and higher Ang2 levels are associated with a poor outcome in patients with sepsis or critical illness [9,20,25,33,34]. Ang1 has anti-inflammatory properties and protects against vascular leakage, while Ang2 promotes inflammation and increases the vascular permeability, leading to the development of ARDS [9,34-36]. Moreover, positive relationships between Ang2 and inflammatory cytokines, such as TNF-alpha and IL-6, have been observed [36]. These results suggest that elevations in Ang2 and a decrease in Ang1 may reflect a pro-inflammatory state and that this is best summarized by the ratio between Ang1 and Ang2 [13,14]. The current study and previous studies suggest that a higher Ang2 level, as well as an imbalance of Ang1 and Ang2 (high Ang2/Ang1 ratio) are both associated with inflammation during critical illness, thus resulting in ALI/ARDS. The administration of Ang1 protects the vasculature from leakage, thereby countering the potentially lethal actions of VEGF and inflammatory agents in animal experiments [37,38]. Calfee et al. reported that the Ang2 levels are affected by the treatment strategies, and that lower Ang2 levels were observed after fluid conservative therapy, which may be beneficial as a result of decreasing the endothelial inflammation in patients with acute lung injury [34]. These results imply that correcting the imbalances between Ang1 and Ang2 by either administering Ang1 or inhibiting Ang2 may represent a new therapeutic strategy for severe inflammatory illnesses, such as ARDS.

Soluble Tie2 (sTie2) is released by the proteolytic cleavage of the extracellular domain by matrix metalloproteases, which occurs constitutively or after VEGF signaling [39]. Cleaved sTie2 binds to both Ang1 and Ang2 to inhibit ligand-mediated Tie2 receptor activation and downstream signaling [40]. The sTie2 levels correlate with the VEGF levels, supporting the *in vivo* shedding of Tie2 through VEGF signaling [41]. The sTie2 levels showed no significant changes between the non-ALI/ARDS groups and ALI/ARDS groups in the present study. sTie2 was previously shown to be higher in septic than in non-septic patients [41]. However, this increase does not rule out a direct role for Ang1 and Ang2 in pulmonary vascular permeability in both septic and non-septic critically ill patients. These results as well as those of a present study suggest that Ang1 and Ang2, especially Ang2, but not sTie2, may be involved in the capillary leakage in ALI/ARDS patients with critical illness.

## Conclusion

This study demonstrated that there were lower sVEGFR2 levels on day 1 and higher sVEGFR1 and Ang2 levels, and an increased Ang2/Ang1 ratio during the entire study period in patients with ALI/ARDS associated with critical illness, including sepsis, severe trauma, and PCAS. sVEGFR2 and Ang2 were found to be independent predictors of the development of ALI/ARDS in critically ill patients. In addition, sVEGFR2 and Ang2 independently predict the 28-day mortality in ALI/ARDS patients associated with critical illness. It should be noted that not only Ang2, but also sVEGFR2, both play a pivotal role in the development of ALI/ARDS in cases of critical illness and can predict the 28-day mortality in ALI/ARDS patients.

## Abbreviations

ALI: Acute lung injury; ARDS: Acute respiratory distress syndrome; VEGF: Vascular endothelial growth factor; VEGFR: Vascular endothelial growth factor receptor; Ang: Angiopoietin; PCAS: Post-cardiac arrest syndrome; APACHE: Acute physiology and chronic health evaluation; SOFA: Sequential organ failure assessment; SIRS: Systemic inflammatory response syndrome; ISS: Injury severity score; max: Maximum score; min: Minimum score.

## Competing interests

The authors declare that they have no competing interests.

## Authors’ contributions

TW analyzed the results, drew the diagrams and wrote the manuscript. SJ had the initial idea, established the immunoassays, performed and supervised the experiments and reviewed the manuscript. SG had the initial idea, designed and supervised the research, identified patients, collected samples, provided clinical data and reviewed the manuscript. AM provided clinical data and reviewed the manuscript. SNS and SZ established the experiments. YY and HY reviewed the manuscript. All authors read and approved the final manuscript.
